# The *MdSLR1b*‐*MdKNOX15*‐*MdGA20ox2*/*MdGA2ox7* module regulates dwarfing by mediating the gibberellin pathway in apple

**DOI:** 10.1111/tpj.71137

**Published:** 2026-09-19

**Authors:** Rui Yan, Xinfeng Zeng, Yuan Wang, Ning Wang, Yilin Yang, Bingyi Liang, Xuemei Zhang, Guohui Qi, Peng Jia

**Affiliations:** ^1^ College of Forestry Hebei Agricultural University Baoding Hebei China; ^2^ Hebei Provincial Key Laboratory of Forest Tree Germplasm Resources and Forest Protection Baoding 071000 China; ^3^ State Key Laboratory of North China Crop Improvement and Regulation Hebei Agricultural University Baoding 071000 China

**Keywords:** apple, dwarf, gibberellin, KNOX, DELLA

## Abstract

Dwarf and high‐density cultivation is a major trend in the apple industry, yet the regulatory mechanisms remain largely unexplored. In this study, we identified *MdKNOX15*, a KNOX transcription factor highly expressed in spur‐type and dwarfing rootstock varieties, as a critical regulator of plant height. Overexpression of *MdKNOX15* caused a gibberellin (GA)‐deficient dwarf phenotype, whereas VIGS‐mediated knockdown increased endogenous GA content and plant height. Exogenous GA alleviated, and uniconazole exacerbated, the dwarfism. Using DAP‐seq and RNA‐seq, we identified *MdGA2ox7* and *MdGA20ox2* as direct target genes. MdKNOX15 activated *MdGA2ox7* and repressed *MdGA20ox2* by binding to (T)TGAC elements in their promoters. Functional assays confirmed that *MdGA2ox7* negatively, and *MdGA20ox2* positively, regulates apple height. Furthermore, the DELLA homolog MdSLR1b physically interacted with MdKNOX15. Overexpression of *MdSLR1b* reduced plant height, while its knockdown alleviated the dwarfism induced by *MdKNOX15* overexpression. MdKNOX15 enhanced MdSLR1b protein stability, especially under GA treatment, and MdSLR1b amplified the transcriptional regulation of downstream genes by MdKNOX15. Collectively, we propose a molecular model in which the *MdKNOX15*–*MdSLR1b*–*MdGA2ox7*/*MdGA20ox2* module governs apple plant height, providing a theoretical basis for breeding dwarf apple varieties.

## Significance Statement

MdKNOX15 promotes apple dwarfing by directly repressing the GA biosynthesis gene *MdGA20ox2* and activating the GA catabolism gene *MdGA2ox7*, while also stabilizing the DELLA protein MdSLR1b to enhance GA signaling inhibition. This dual mechanism reduces bioactive GA levels and suppresses stem elongation.

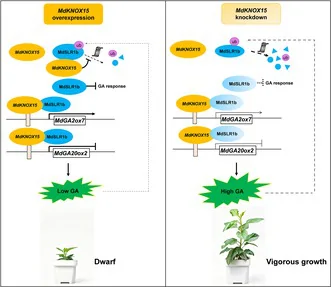

Dwarfing is a key trait for modern apple orchards, but the underlying regulatory network remains largely elusive. We uncover a *MdSLR1b*–*MdKNOX15*–*MdGA20ox2*/*MdGA2ox7* module that fine‐tunes gibberellin homeostasis, revealing how SLR1b and KNOX proteins coordinate hormone metabolism and response to control plant height and offering a molecular target for breeding dwarf apple cultivars.

## INTRODUCTION

Plant height is a key agronomic trait affecting crop yield, lodging resistance, planting density, and field management efficiency. It is influenced by external environmental factors (e.g., light and temperature) and endogenous nutrient levels (e.g., nitrogen). The “Green Revolution” of the 20th century was largely attributed to the widespread adoption of semi‐dwarf alleles in wheat and rice, which reduced stem elongation and allocated more photosynthetic products to grains, thereby greatly increasing cereal yields. The genetic basis of this revolution lies in loss‐of‐function mutations in gibberellin (GA) metabolism or signaling pathways: the rice *sd1* mutation affects GA20oxidase (a GA biosynthetic enzyme), causing GA deficiency (Sasaki et al., 2002); the wheat *Rht* (Reduced height) mutants (e.g., *Rht‐B1b*/*Rht‐D1b*) encode mutated DELLA proteins that cannot be degraded after GA perception, resulting in GA‐insensitive dwarfism (Peng et al., 1999). These two mutation types represent the two classic molecular mechanisms regulating plant height —reduced GA synthesis and GA signal insensitivity, and together laid the genetic foundation for the Green Revolution. These milestone discoveries established GA as a central regulator of plant height and revealed the great potential of manipulating the GA pathway for crop improvement.

Plant height is regulated by complex interactions between endogenous hormone networks and environmental signals. Many plant hormones have been shown to be associated with plant height, such as auxin, brassinosteroids, and cytokinins (Miao et al., 2024). Among plant hormones, GAs play a particularly prominent role in promoting stem elongation, cell division, and internode growth. GA biosynthesis involves multiple enzymatic steps, with GA20oxidase (GA20ox) and GA3oxidase (GA3ox) catalyzing the final steps to produce bioactive GAs, while GA2oxidase (GA2ox) inactivates GAs through catabolism (Hedden & Phillips, 2000). The expression levels of GA synthesis and metabolism genes are finely tuned, thereby affecting endogenous bioactive GA content and plant height (Yamaguchi, 2008). Moreover, exogenous GA application promotes internode elongation and increases plant height in many species, whereas GA biosynthesis inhibitors cause dwarf phenotypes (Nagai et al., 2020), further confirming the central role of GA in height regulation.

The GA signaling pathway operates through a derepression mechanism centered on DELLA family transcriptional regulators (Gomez et al., 2020; Gómez et al., 2019). In the absence of bioactive GA, DELLA proteins (e.g., GAI, RGA, RGL in Arabidopsis; SLR1 in rice; RHT in wheat) act as growth repressors by interacting with various transcription factors and co‐regulators to suppress gene expression. When GA is perceived by the GID1 receptor, conformational changes occur, promoting the formation of the GA‐GID1‐DELLA complex, which then ubiquitinates DELLA proteins and targets them for degradation via the 26S proteasome, thereby relieving growth inhibition (Dahal et al., 2025). DELLA proteins are highly conserved across the plant kingdom, and loss‐of‐function mutations cause constitutive GA responses and excessive elongation. For example, the *slr1* (slender rice) mutant exhibits an elongated phenotype even in the presence of GA synthesis inhibitors (Ikeda et al., 2001). Conversely, stabilized DELLA variants (e.g., Arabidopsis *gai* and *rga* mutants) lead to severe dwarfism and reduced GA sensitivity (Nohales & Kay, 2019), indicating that DELLA‐mediated growth restraint is a fundamental determinant of plant height.

Multiple transcription factors integrate hormonal and developmental signals to coordinate shoot architecture and meristem activity. KNOTTED1‐like homeobox (KNOX) transcription factors constitute a family of homeodomain proteins that are key regulators of shoot apical meristem (SAM) maintenance, leaf morphogenesis, and organ patterning (Jia et al., 2023). Since the discovery of the *Knotted‐1* gene in maize over 30 years ago (Vollbrecht et al., 1991), numerous studies have shown that *KNOX* genes are indispensable for meristem function. Extensive research in model plants has revealed that KNOX transcription factors exert their pleiotropic effects through multiple downstream pathways, including direct regulation of GA metabolism in the SAM and preventing premature differentiation (Jasinski et al., 2005; Nagasaki et al., 2001).

Apple (*Malus domestica* Borkh.) is one of the four major fruit tree species worldwide, and breeding compact, dwarf, or semi‐dwarf varieties has long been a core objective of breeding programs, owing to the advantages of high‐density planting, reduced pruning costs, and increased orchard productivity. Despite the well‐documented importance of KNOX in many plant species, their functional roles in woody perennials, particularly in regulating apple tree architecture and plant height, remain poorly understood. Although we have found that heterologous overexpression of *MdKNOX15* may inhibit plant height (Jia, Xing, Zhang, Chen, et al., 2021), its role in regulating apple plant height has not been systematically investigated. In this study, we aimed to clarify the functional role of the MdKNOX15 transcription factor in regulating plant height, focusing on its interplay with the GA pathway. By integrating gene expression analysis, transgenic approaches, and molecular interaction studies, we propose a molecular mechanism by which the *MdSLR1b*‐*MdKNOX15*‐*MdGA2ox7*/*MdGA20ox2* module regulates apple plant height. Our findings are expected to provide new insights into the molecular regulation of apple tree architecture, and offer potential genetic targets for breeding dwarf apple varieties adapted to modern high‐density orchard systems.

## RESULTS

### *MdKNOX15* is highly expressed in spur‐type apple varieties and dwarfing rootstocks

**Figure 1:**
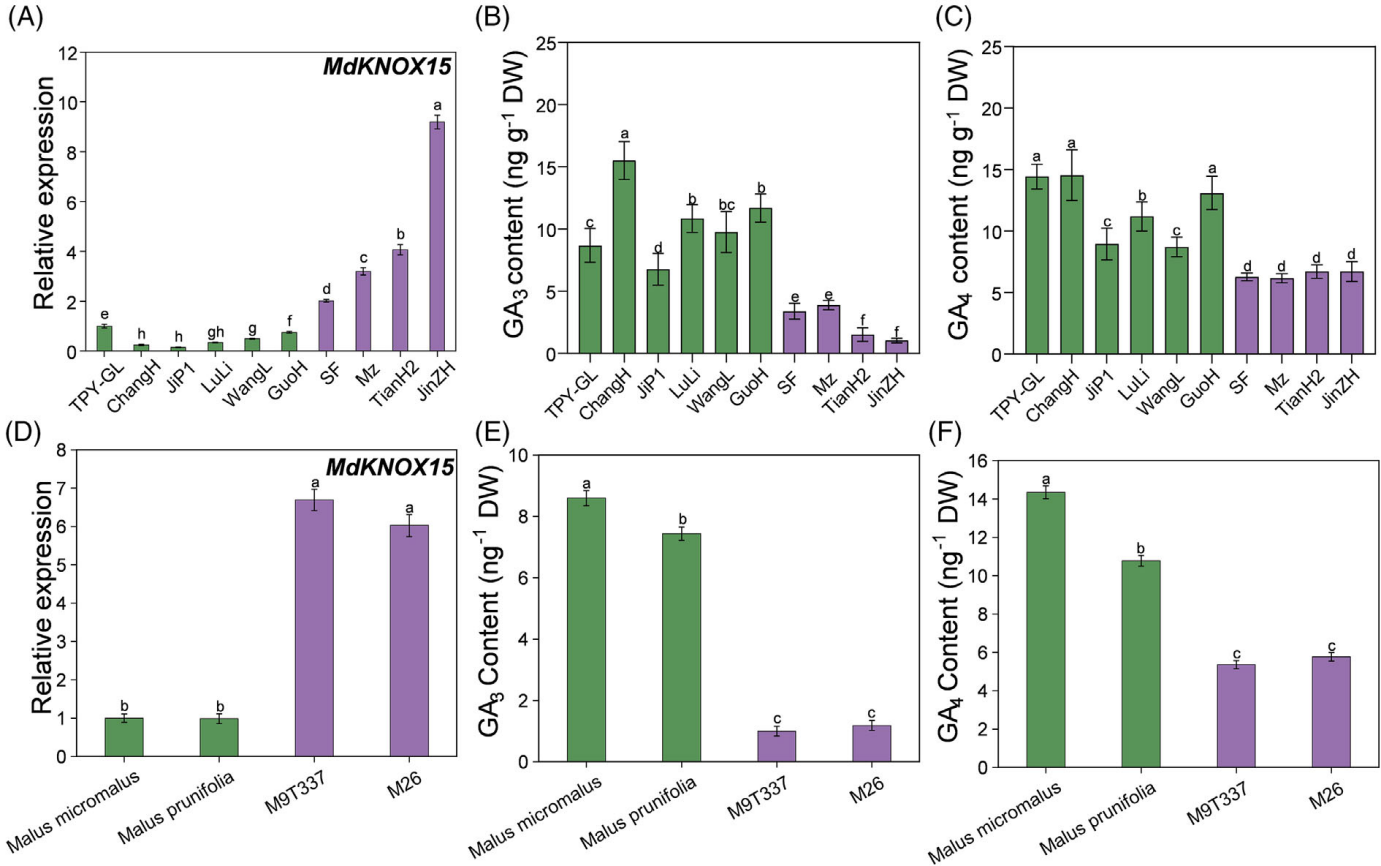
Relative expression levels of *MdKNOX15* and endogenous GA contents in different apple varieties. (A) Relative expression of *MdKNOX15* in shoot tips of standard‐type and spur‐type apple varieties. *MdActin* was used for normalization. The expression level in “Pacific Gala” was set to 1. (B) GA_3_ content in shoot tips of standard‐type and spur‐type varieties. (C) GA_4_ content in shoot tips of standard‐type and spur‐type varieties. Six standard‐type apple varieties (“Pacific Gala,” TPY‐GL; “Changhong,” ChangH; “Jiping 1,” JiP1; “Luli”; “,” WangL; “Guohong,” GuoH) and four spur‐type varieties (“Shifu Spur,” SF; “Miyazaki Spur,” Mz; “Tianhong 2,” TianH2; “Jinzhouhong,” JinZH) were used. (D) *MdKNOX15* expression in dwarfing rootstocks (M9T337, M26) and standard rootstocks (*Malus micromalus*, *Malus prunifolia*). *MdActin* was used for normalization. The expression level in *M. micromalus* was set to 1. (E) GA_3_ content in dwarfing and standard rootstocks. (F) GA_4_ content in dwarfing and standard rootstocks. Data are means ± SD of three independent experiments. The significance test was carried out by one‐way analysis of variance (ANOVA) followed by Tukey's test, and different lowercase letters above the bar charts indicate significant differences between apple varieties at the level of *P* < 0.05.

Spur‐type apple varieties possess a higher ratio of spur shoots to long shoots compared with standard‐type varieties. To verify whether *MdKNOX15* is involved in regulating apple shoot growth, we compared the relative expression levels of *MdKNOX15* in six standard‐type apple varieties (“Pacific Gala,” TPY‐GL, a red peel sport of “Royal Gala”; “Changhong,” ChangH, an early‐ripening bud sport of “Red Fuji”; “Jiping 1,” JiP1, a new early‐maturing apple cultivar selected from open‐pollinated seedlings of “Mato”; “Luli,” a hybrid cultivar derived from “Mato” × “Royal Gala”; “Wanglin,” WangL, traditionally called “Orin”; “Guohong,” GuoH, a hybrid cultivar derived from “Ralls Janet” × “Starking”) and four spur‐type varieties (“Shifu Spur,” SF, a bud sport of “Nagafu No. 2”; “Miyazaki Spur,” Mz, a bud sport of “Fuji”; “Tianhong 2,” TianH2, a variant of “Red Fuji”; “Jinzhouhong,” JinZH, a bud sport of “Meiguihong”) (Figure S1). The results showed that *MdKNOX15* expression was significantly higher in spur‐type varieties than in standard‐type varieties (Figure 1A). We also compared its expression in two dwarfing rootstocks (M9T337, M26) and two standard rootstocks (*Malus micromalus* and *Malus prunifolia*) and found that *MdKNOX15* expression was significantly higher in dwarfing rootstocks than in standard rootstocks (Figure 1D). Endogenous GA content analysis showed that GA_3_ and GA_4_ levels were significantly higher in standard‐type varieties than in spur‐type varieties (Figure 1B,C), and were significantly lower in dwarfing rootstocks than in standard rootstocks (Figure 1E,F). These results suggest that *MdKNOX15* may be involved in regulating shoot length, and that this regulation may be associated with GA content.

### Overexpression of *MdKNOX15* causes GA deficiency and a dwarf phenotype

**Figure 2:**
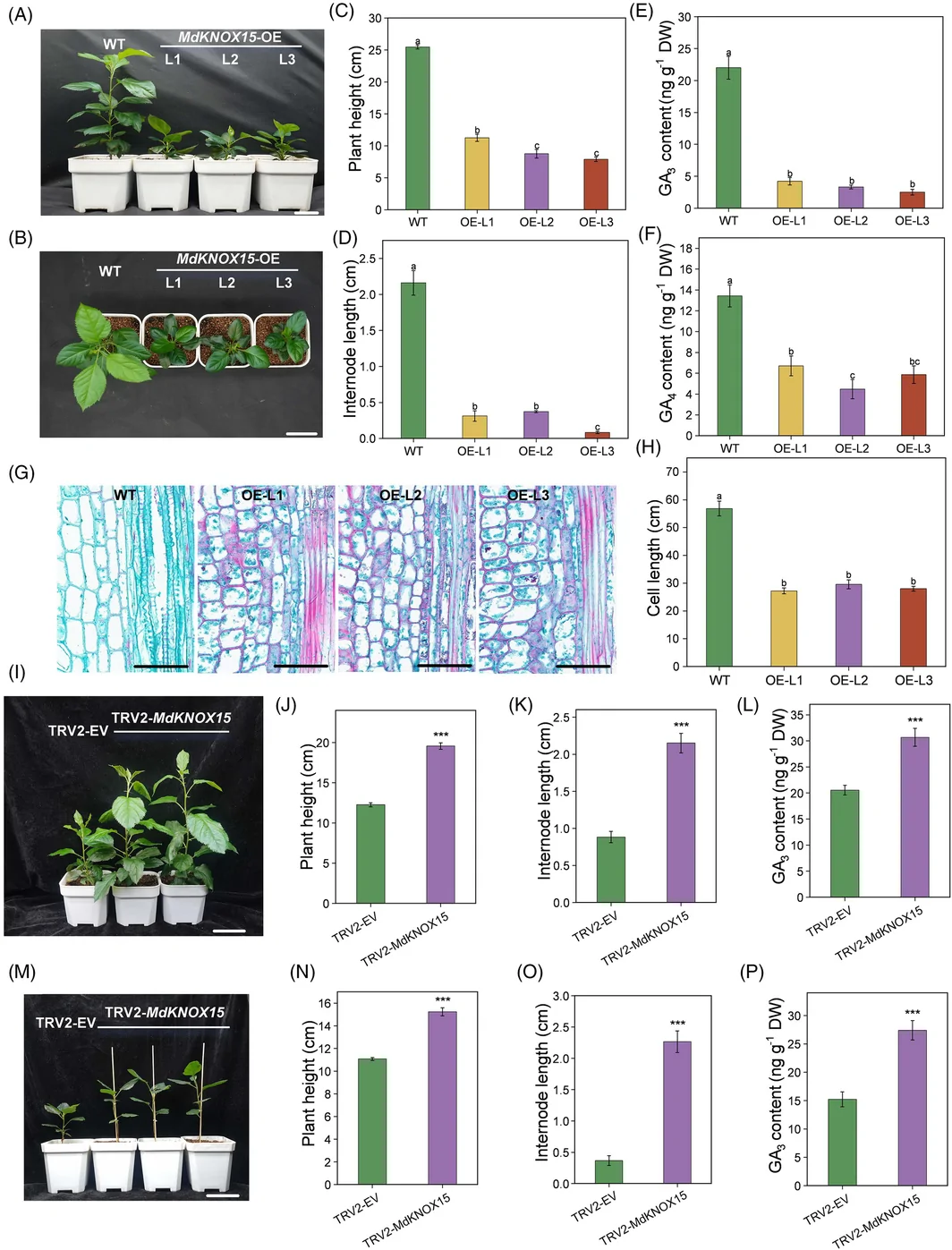
MdKNOX*15* suppresses GA accumulation and causes dwarfing in apple. (A–H) MdKNOX15 overexpression inhibits apple growth and GA accumulation. (A, B) Phenotypes of 2‐month‐old MdKNOX15‐OE apple seedlings: (A) top view, (B) side view. (C) Plant height. (D) Internode length. (E) GA3 content. (F) GA4 content. (G, H) Longitudinal stem section observation (G) and internode cell length (H) of transgenic and WT plants. (I, P) MdKNOX15 knockdown promotes apple growth and GA accumulation. (I, L) Phenotypic analysis of MdKNOX15 knockdown in “GL‐3”: (I) photographs of 25‐day‐old seedlings, (J) plant height, (K) internode length, (L) GA3 content. (M–P) Phenotypic analysis of MdKNOX15 knockdown in M. hupehensis: (M) photographs of 2‐month‐old seedlings, (N) plant height, (O) internode length, (P) GA3 content. Scale bars = 5 cm for plant photographs; for sections, bar = 100 μm. Data are mean ± SD of three independent experiments. Statistical significance was determined using Student's *t*‐test for comparisons between TRV2‐EV and TRV2‐MdKNOX15 plants, and asterisks (*) above the bar charts denote significant differences (*P < 0.05; **P < 0.01; ***P < 0.001). One‐way analysis of variance (ANOVA) followed by Tukey's test was used for multiple comparisons, and different lowercase letters above the bar charts indicate significant differences among WT and various MdKNOX15‐OE transgenic plants (*P* < 0.05).

To elucidate the function of MdKNOX15 in regulating apple growth, we overexpressed *MdKNOX15* in “GL‐3,” an open‐pollinated line of “Royal Gala” with high regeneration capacity and sensitivity to *Agrobacterium* (Dai et al., 2013). PCR and Western blot analyses confirmed three independent transgenic lines, in which *MdKNOX15* expression was significantly higher than in the wild‐type (WT) (Figure S2a–d). The *MdKNOX15* overexpression (OE) lines exhibited a clear dwarf phenotype (Figure 2A–C), with average plant height significantly reduced to 30–40% of that of the WT (Figure 2C). The internode length of *MdKNOX15*‐OE lines was also reduced (Figure 2D). Histological analysis showed that cell length in longitudinal stem sections of the *MdKNOX15*‐OE lines was shortened (Figure 2G,H), suggesting that MdKNOX15 negatively regulates plant height. Because plant hormones are well known to regulate plant architecture, we examined whether MdKNOX15 affects hormone homeostasis, especially hormones related to shoot elongation. Hormone content assays showed no significant changes in the levels of auxin, brassinosteroids, and cytokinins (Figure S3); however, the endogenous GA_3_ and GA_4_ levels in *MdKNOX15*‐OE lines were significantly lower than those in the WT (Figure 2E,F). Therefore, MdKNOX15 may regulate plant height by affecting endogenous GA content.

To further verify the function of MdKNOX15, we used virus‐induced gene silencing (VIGS) to generate *MdKNOX15* knockdown plants in apple “GL‐3” and *Malus hupehensis* (*M. hupehensis*, Pingyi Tiancha). Compared with the empty vector control (TRV2‐EV), TRV2‐*MdKNOX15* (*MdKNOX15* knockdown) plants showed an approximately 50% reduction in *MdKNOX15* transcript levels (Figure S2e,f). These knockdown plants exhibited significantly promoted growth, with average plant height about twice that of the control (Figure 2I,J,M,N), and internode length also significantly increased (Figure 2K,O). Moreover, endogenous GA_3_ levels were significantly elevated in the knockdown plants (Figure 2L,P). Collectively, these results indicate that MdKNOX15 may cause dwarfing by suppressing endogenous GA accumulation.

### GA treatment partially rescues the dwarf phenotype of *MdKNOX15* transgenic plants

**Figure 3:**
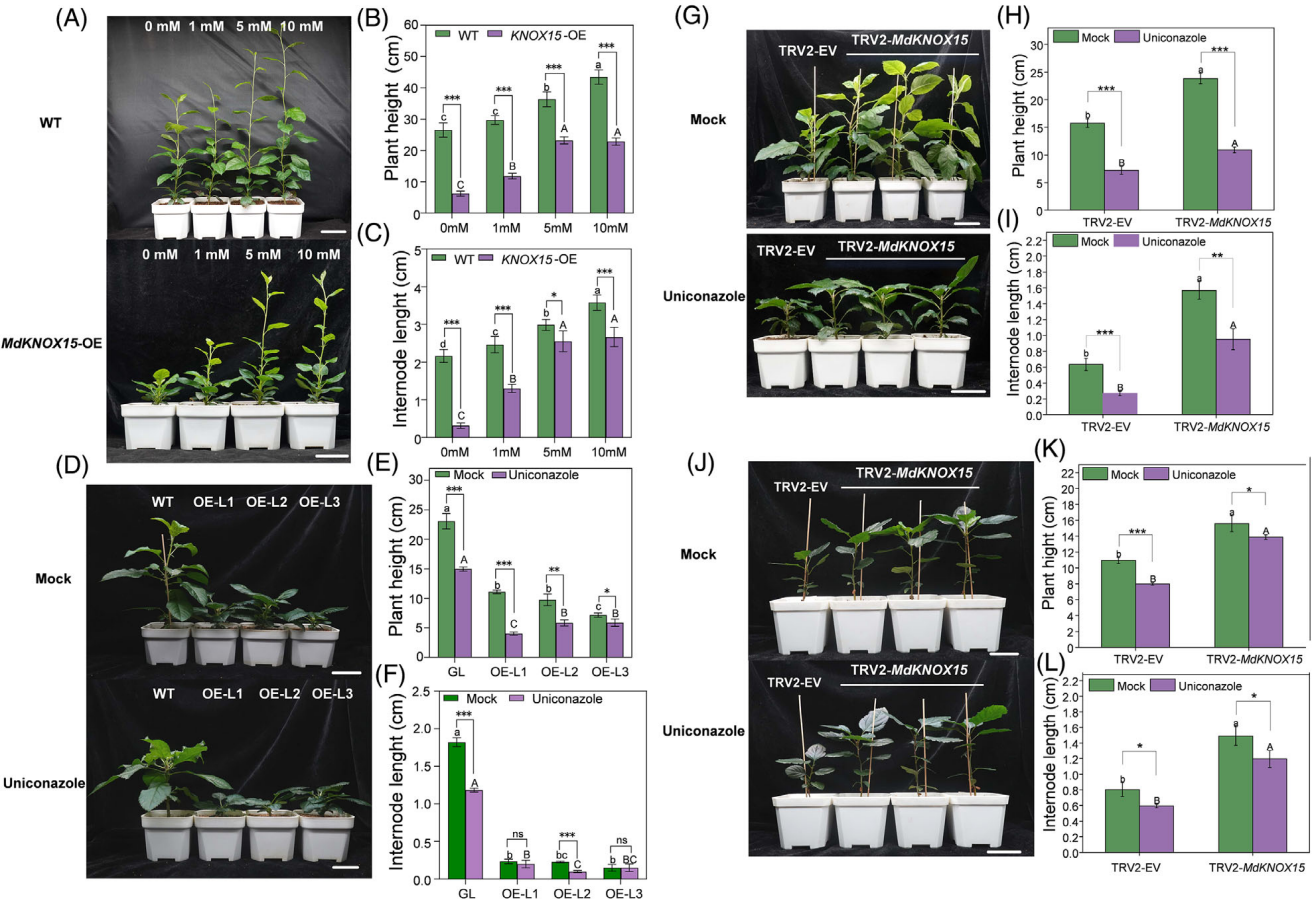
Exogenous GA treatment rescues, whereas uniconazole exacerbates, the dwarf phenotype of *MdKNOX15*‐OE apple plants. (A–C) Exogenous GA3 spray promotes growth of MdKNOX15‐OE (L3) transgenic apple plants. (A) Phenotypic photographs after GA3 treatment at different concentrations. (B) Plant height. (C) Internode length. In figures (B) and (C), asterisks (*) above the bars indicate significant differences between WT and transgenic plants at the same concentration (*P < 0.05; **P < 0.01; ***P < 0.001); different uppercase and lowercase letters above the bars indicate significant differences among different concentrations within the same line at the *P* < 0.05 level. (D–F) Exogenous uniconazole (UCZ) spray further inhibits growth of MdKNOX15‐OE plants. (D) Phenotypic photographs after UCZ treatment. (E) Plant height. (F) Internode length. (G–I) Exogenous UCZ spray inhibits growth of MdKNOX15 knockdown “GL‐3” plants. (G) Phenotypic photographs. (H) Plant height. (I) Internode length. (J–L) Exogenous UCZ spray inhibits growth of MdKNOX15 knockdown *M. hupehensis* plants. (J) Phenotypic photographs. (K) Plant height. (L) Internode length. Scale bars = 5 cm. Data are mean ± SD of three independent experiments. In figures (E), (F), (H), (I), (K), and (L), asterisks (*) above the bars indicate significant differences between treatment and mock within the same line (*P < 0.05; **P < 0.01; ***P < 0.001); different uppercase (for KNOX15‐OE) and lowercase (for WT) letters above the bars represent significant differences among different lines under the same treatment condition at the *P* < 0.05 level. Student's *t*‐test was used for comparisons between two groups. One‐way ANOVA followed by Tukey's test was applied for comparisons among multiple groups (*P* < 0.05).

To further verify the effect of altered GA levels on the plant height, different concentrations of exogenous GA_3_ were sprayed onto both the WT and *MdKNOX15*‐OE line (L3). GA_3_ treatment partially restored the plant height and internode length of *MdKNOX15*‐OE plants, with visible new shoot elongation at the apex (Figure 3A–C). As the exogenous GA_3_ concentration increased, the difference in plant height between *MdKNOX15*‐OE and the WT progressively narrowed, although the recovery did not reach WT levels. Conversely, application of uniconazole (UCZ), a GA biosynthesis inhibitor, to *MdKNOX15*‐OE plants caused severe growth inhibition and further exacerbated the dwarf phenotype (Figure 3D–F). Similarly, UCZ treatment significantly suppressed the increased plant height phenotype of *MdKNOX15* knockdown “GL‐3” (Figure 3G,H) and *M. hupehensis* (Figure 3J,K) lines, with internode length markedly shortened compared with the mock treatment (Figure 3I,L). These results further confirm that MdKNOX15 causes apple dwarfing by inhibiting endogenous GA accumulation.

### MdKNOX15 targets genes encoding GA catabolic and biosynthetic enzymes

**Figure 4:**
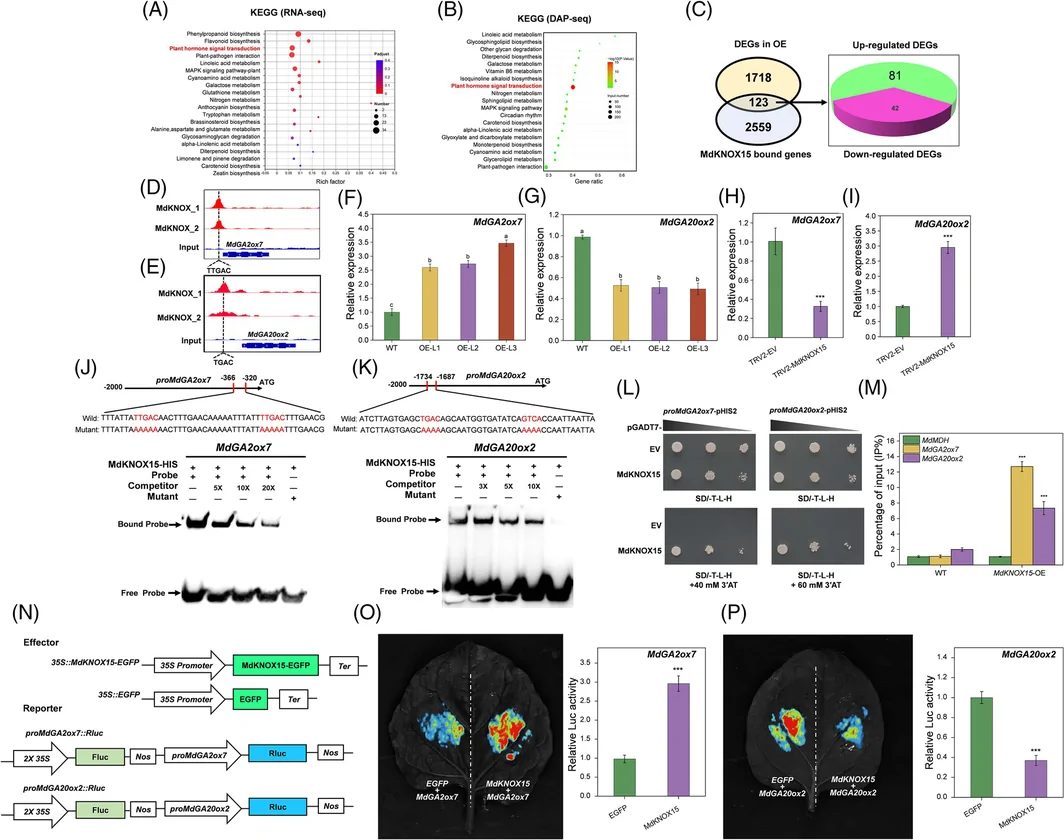
MdKNOX15 regulates gibberellin biosynthetic and catabolic enzyme genes. (A–C) Identification of downstream target genes of MdKNOX15 by integrating RNA‐seq and DAP‐seq. (A) KEGG analysis of DEGs in *MdKNOX15*‐OE versus WT. (B) KEGG analysis of genes bound by MdKNOX15 in DAP‐seq. (C) Venn diagram showing overlapping genes between RNA‐seq and DAP‐seq. (D, E) Promoter binding peaks and motif analysis for *MdGA2ox7* (D) and *MdGA20ox2* (E). (F, G) Relative expression levels of *MdGA2ox7* (F) and *MdGA20ox2* (G) in *MdKNOX15*‐OE “GL‐3” apple, with WT “GL‐3” as control. (H, I) Relative expression levels of *MdGA2ox7* (H) and *MdGA20ox2* (I) in *MdKNOX15* knockdown apple, with empty vector (TRV2‐EV) control. (J, K) EMSA showing binding of MdKNOX15 to the promoters of *MdGA2ox7* (J) and *MdGA20ox2* (K). Wild‐type and mutant probes were biotin‐labeled; competitor probes were unlabeled (same sequence as wild‐type). Red letters indicate the wild‐type (T)TGAC motif and the mutant motif. (L) Yeast one‐hybrid (Y1H) assay showing that MdKNOX15 binds to the promoters of *MdGA2ox7* (left) and *MdGA20ox2* (right) in yeast cells. (M) ChIP‐qPCR results showing the binding of MdKNOX15 to the promoters of *MdGA2ox7* and *MdGA20ox2*. *MDH* was used as a negative control and for standardization. (N–P) Dual‐luciferase reporter assays showing the effect of MdKNOX15 on the promoter activities of *MdGA2ox7* and *MdGA20ox2*. (N) Schematic diagram of effector (MdKNOX15) and reporter (*MdGA2ox7* and *MdGA20ox2*) constructs. (O) Luciferase signal and ratio showing that MdKNOX15 activates the *MdGA2ox7* promoter. (P) Luciferase signal and ratio showing that MdKNOX15 represses the *MdGA20ox2* promoter. For (O) and (P), the Rluc/Fluc ratio from the empty effector plus respective promoter was set to 1. Data are mean ± SD of three independent experiments. Statistical significance was determined using Student's *t*‐test for comparisons between two groups. For (H) and (I), asterisks (*) above the bar charts represent significant differences between TRV2‐EV and TRV2‐*MdKNOX15* plants. For (M), asterisks (*) above the bar charts represent significant differences between WT and *MdKNOX15*‐OE plants (same gene in ChIP‐qPCR). For (O) and (P), asterisks (*) above the bar charts represent significant differences between EGFP and MdKNOX15 effectors (*P < 0.05; **P < 0.01; ***P < 0.001). One‐way analysis of variance (ANOVA) followed by Tukey's test was used for multiple comparisons in (F) and (G), and different lowercase letters above the bar charts indicate significant differences among WT and various *MdKNOX15*‐OE transgenic plants (*P* < 0.05).

To gain a deeper understanding of the molecular mechanism by which MdKNOX15 causes dwarfing, we performed RNA‐sequencing (RNA‐seq) on shoot tips of WT and *MdKNOX15*‐OE plants. Compared with the WT, we identified 1328 upregulated and 513 downregulated genes (Figure S4). KEGG pathway analysis showed that these differentially expressed genes (DEGs) were enriched in the plant hormone signal transduction pathway (Figure 4A). To determine the genome‐wide DNA‐binding sites and direct target genes of MdKNOX15, we performed DNA affinity purification sequencing (DAP‐seq). Kyoto encyclopedia of genes and genomes (KEGG) pathway analysis of the target genes also revealed enrichment in the plant hormone signal transduction pathway (Figure 4B). Integrating the RNA‐seq and DAP‐seq data, we identified 123 common genes (Figure 4C), including the GA catabolic enzyme gene *MdGA2ox7* and the GA biosynthetic enzyme gene *MdGA20ox2*. Peak sequence analysis showed that the promoter regions of both genes contain (T)TGAC motifs (Figure 4D,E). RT‐qPCR analysis revealed that *MdGA2ox7* expression was increased in *MdKNOX15*‐OE lines, whereas *MdGA20ox2* expression was significantly decreased (Figure 4F,G). Conversely, *MdKNOX15* knockdown led to downregulation of *MdGA2ox7* and upregulation of *MdGA20ox2* (Figure 4H,I), suggesting that MdKNOX15 may bind to the promoters of *MdGA2ox7* and *MdGA20ox2* and affect their expression.

We further validated the specific interaction between MdKNOX15 and the *MdGA2ox7* promoter using EMSA *in vitro*. The MdKNOX15‐HIS protein specifically bound to the *MdGA2ox7* promoter probe, and this binding was competitively inhibited by an excess of unlabeled probe, confirming a direct and specific interaction (Figure 4J). When the (T)TGAC motif was mutated, the binding was abolished, indicating that MdKNOX15 recognizes this motif to bind the *MdGA2ox7* promoter. EMSA also confirmed the specific interaction between MdKNOX15 and the *MdGA20ox2* promoter (Figure 4K). Y1H assays were performed to further identify the interaction between MdKNOX15 and the *MdGA2ox7/MdGA20ox2* promoters. The yeast strains co‐transformed with the MdKNOX15‐pGADT7 and pHIS2‐*proMdGA2ox7* (or pHIS2‐*proMdGA20ox2*) vectors grew in the selection medium supplemented with 3‐AT, while the control strains transformed with the pGADT7 empty vectors did not (Figure 4L), indicating the binding ability of MdKNOX15 on the *MdGA2ox7/MdGA20ox2* promoters. In addition, ChIP‐qPCR analyses were performed to identify the binding ability of MdKNOX15 to *MdGA2ox7/MdGA20ox2* promoters *in vivo* (Figure 4M). To determine whether MdKNOX15 directly regulates the transcription of *MdGA2ox7* and *MdGA20ox2*, we performed dual‐luciferase reporter assays using MdKNOX15 as an effector and the *MdGA2ox7* or *MdGA20ox2* promoter driving a luciferase reporter gene (Figure 4N). Quantitative analysis showed that the Rluc/Fluc ratio was significantly increased in samples co‐transformed with 35S::MdKNOX15‐EGFP and the *MdGA2ox7* promoter compared with the 35S::EGFP control (Figure 4O), whereas the ratio was significantly decreased with the *MdGA20ox2* promoter (Figure 4P). These results demonstrate that MdKNOX15 directly binds to the promoters of both genes and regulates their transcription.

RT‐qPCR results showed that *MdGA2ox7* was highly expressed in spur‐type varieties and dwarfing rootstocks (Figure S5a,c), whereas *MdGA20ox2* was highly expressed in standard‐type varieties and standard rootstocks (Figure S5b,d), suggesting that *MdGA2ox7* negatively and *MdGA20ox2* positively regulates plant height. To further verify the roles of these genes in apple plant height, we overexpressed or silenced them using *Agrobacterium*‐mediated transformation. Previous results have shown that heterologous overexpression of *MdGA2ox7* significantly inhibits plant height in transgenic Arabidopsis (Yan et al., 2024); here, *MdGA2ox7* knockdown in apple promoted plant height, accompanied by an increase in GA content (Figure S6a–f). Meanwhile, *MdGA20ox2* overexpression in Arabidopsis significantly increased plant height and internode length (Figure S7a–d), whereas *MdGA20ox2* knockdown in apple suppressed plant height and internode length (Figure S7e–h). In addition, the endogenous GA levels in the *MdGA20ox2* knockdown plants were significantly reduced (Figure S7i,j), and exogenous GA treatment partially rescued the height and internode length of the *MdGA20ox2* knockdown lines (Figure S7k–m). Taken together, these data indicate that MdKNOX15 directly targets *MdGA2ox7* and *MdGA20ox2* and thereby causes apple dwarfing.

### MdKNOX15 interacts with and stabilizes MdSLR1b

**Figure 5:**
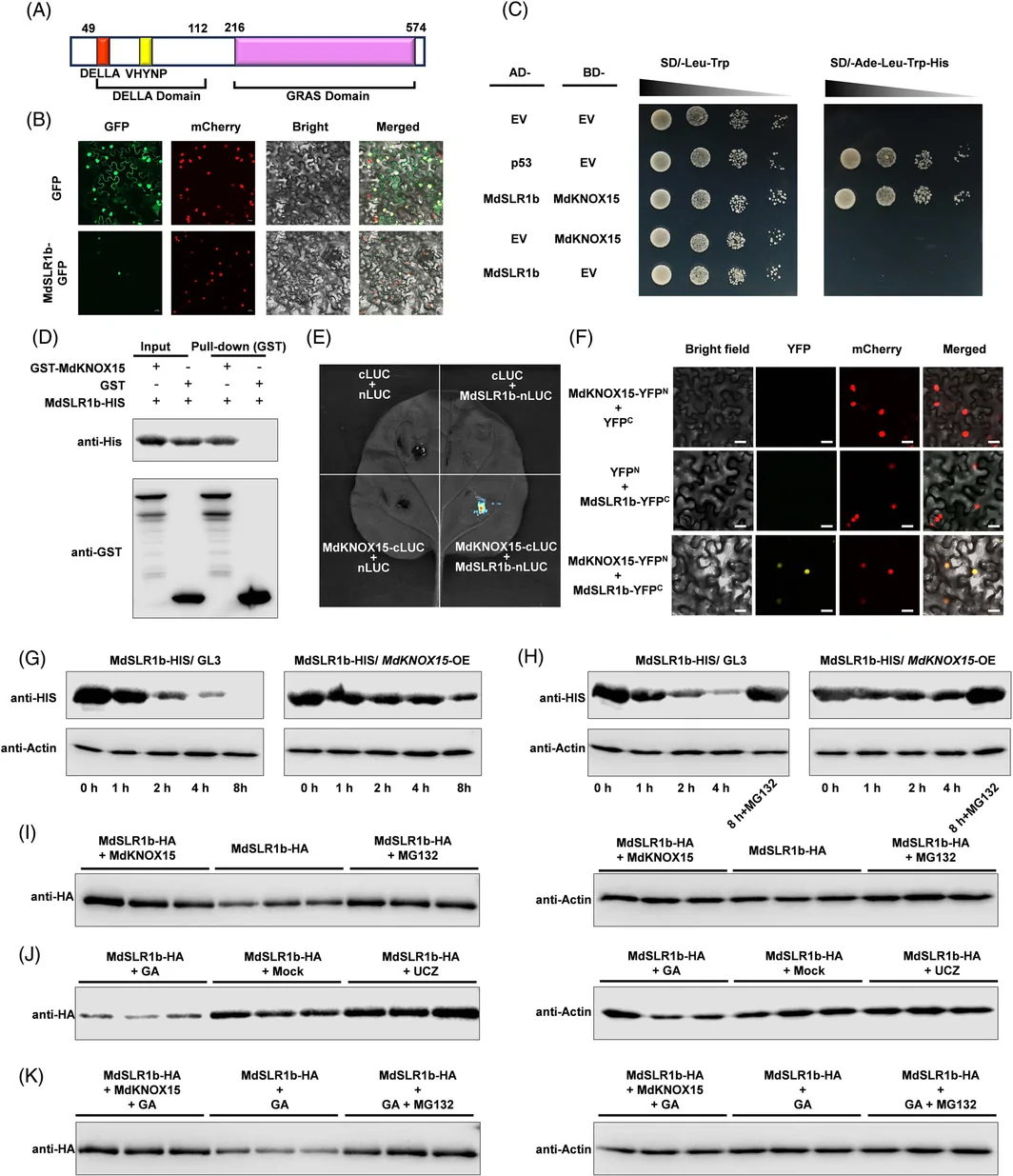
MdKNOX15 interacts with and stabilizes MdSLR1b. (A–F) Interaction between MdKNOX15 and MdSLR1b. (A) Domain structure of MdSLR1b. (B) Subcellular localization of MdSLR1b in tobacco leaves. (C) Y2H assay showing interaction in yeast. AD‐p53 was used as a positive control, and transformants were grown on SD/−Trp/−Leu medium and SD/−Ade/−Trp/−Leu/−His medium, as presented. (D) GST pull‐down assay showing *in vitro* interaction. (E) Luciferase complementation assay (LCA) showing interaction in tobacco leaves. (F) BiFC assay showing interaction in tobacco leaf nuclei. (G–K) Effect of MdKNOX15 on MdSLR1b protein stability. (G, H) MdKNOX15 maintains MdSLR1b protein stability *in vitro*. Proteins extracted from transgenic apple leaves (WT “GL‐3” or *MdKNOX15*‐OE) were incubated with MdSLR1b‐HIS protein. Samples were harvested at indicated time points. Actin served as a loading control. For “8 h + MG132” in (H), 50 μM MG132 was added at the start of incubation. (I) MdKNOX15 maintains MdSLR1b protein stability *in vivo*. MdSLR1b‐HA was transiently expressed alone or together with MdKNOX15‐FLAG in tobacco. For MG132 intervention, leaves were infiltrated with 25 μM inhibitor solution 16 h before sampling. (J) Effect of GA and UCZ on MdSLR1b stability. Leaves were sprayed with 5 mM GA or 100 μM UCZ 24 h before sampling. (K) MdKNOX15 maintains MdSLR1b stability under GA treatment. GA (5 mM) was applied 24 h before sampling; for MG132 intervention, leaves were infiltrated with 25 μM inhibitor solution 16 h before sampling. Actin served as a loading control.

To decipher the regulatory function of MdKNOX15 in plant growth, we performed yeast two‐hybrid (Y2H) screening to capture candidate interactors. A homolog of the DELLA family, which is highly similar to OsSLR1 from rice (Ikeda et al., 2001), was identified and designated MdSLR1b (Figure S8). Sequence analysis showed that MdSLR1b contains a DELLA domain (including the canonical DELLA and VHYPN sequences) at its N‐terminus and a large GRAS domain at its C‐terminus (Figure 5A). Subcellular localization revealed that MdSLR1b is localized to the nucleus (Figure 5B). Y2H point‐to‐point validated that yeast co‐expressed with bait (MdKNOX15) and prey (MdSLR1b) vectors grew normally on quadruple‐dropout (SD/−T‐L‐A‐H) medium, whereas yeast co‐expressed with bait and empty prey vectors did not (Figure 5C), signifying a binding interface between MdKNOX15 and MdSLR1b in yeast. To validate this interaction, *in vitro* GST pull‐down assay was carried out, and the MdSLR1b‐HIS exhibited selective retention by GST‐MdKNOX15 fusion protein but not by GST alone (Figure 5D). Furthermore, a luciferase complementation assay (LCA) in tobacco leaves showed strong luciferase activity only when cLUC‐MdKNOX15 and nLUC‐MdSLR1b were co‐expressed, whereas no activity was detected with the empty controls (Figure 5E), indicating that MdKNOX15 and MdSLR1b interact in plant cells. Bimolecular fluorescence complementation (BiFC) analysis was performed in tobacco leaf cells. Co‐expression of MdKNOX15‐YFP^N^ (N‐terminus of YFP) and MdSLR1b‐YFP^C^ (C‐terminus of YFP) constructs resulted in strong fluorescence signals in the nucleus (Figure 5F), providing direct evidence for their interaction in plant cells.

As DELLA proteins are key regulators of GA signaling and their stability is regulated by multiple factors, we assessed the effect of MdKNOX15 on MdSLR1b protein turnover using a cell‐free degradation assay. Total protein extracts from leaves of *MdKNOX15*‐OE or WT “GL‐3” were co‐incubated with MdSLR1b‐HIS. Proteins were collected at indicated time points, and residual protein levels were monitored by immunoblotting using an anti‐HIS antibody. The results showed that MdSLR1b‐HIS degradation was slowed in the presence of the *MdKNOX15*‐OE protein extract (Figure 5G). When MG132 (a proteasome inhibitor) was added to the reaction, MdSLR1b‐HIS degradation was further inhibited (Figure 5H), suggesting that MdKNOX15 may slow the proteolytic processing of MdSLR1b by interfering with the 26S proteasome pathway. To examine whether MdKNOX15 affects MdSLR1b protein stability *in vivo*, we performed transient co‐expression assays in tobacco leaves. When MdSLR1b‐HA was expressed alone, its protein accumulation was low, but co‐expression with MdKNOX15‐FLAG significantly increased its abundance (Figure 5I). Notably, MG132 treatment also increased the protein level of MdSLR1b‐HA when expressed alone (Figure 5I), indicating that MdKNOX15 may inhibit the 26S proteasome‐mediated degradation of MdSLR1b *in vivo*. To investigate the effect of GA on the stability of the MdSLR1b protein, spraying treatments with GA and UCZ were applied. GA reduced MdSLR1b abundance, whereas UCZ treatment significantly increased it (Figure 5J). Importantly, when MdKNOX15‐FLAG was co‐expressed, MdSLR1b abundance was markedly increased, especially under GA treatment, compared with MdSLR1b expression alone (Figure 5K). Therefore, we conclude that MdKNOX15 enhances the protein stability of MdSLR1b, particularly in the presence of GA.

### MdSLR1b negatively regulates plant height

**Figure 6:**
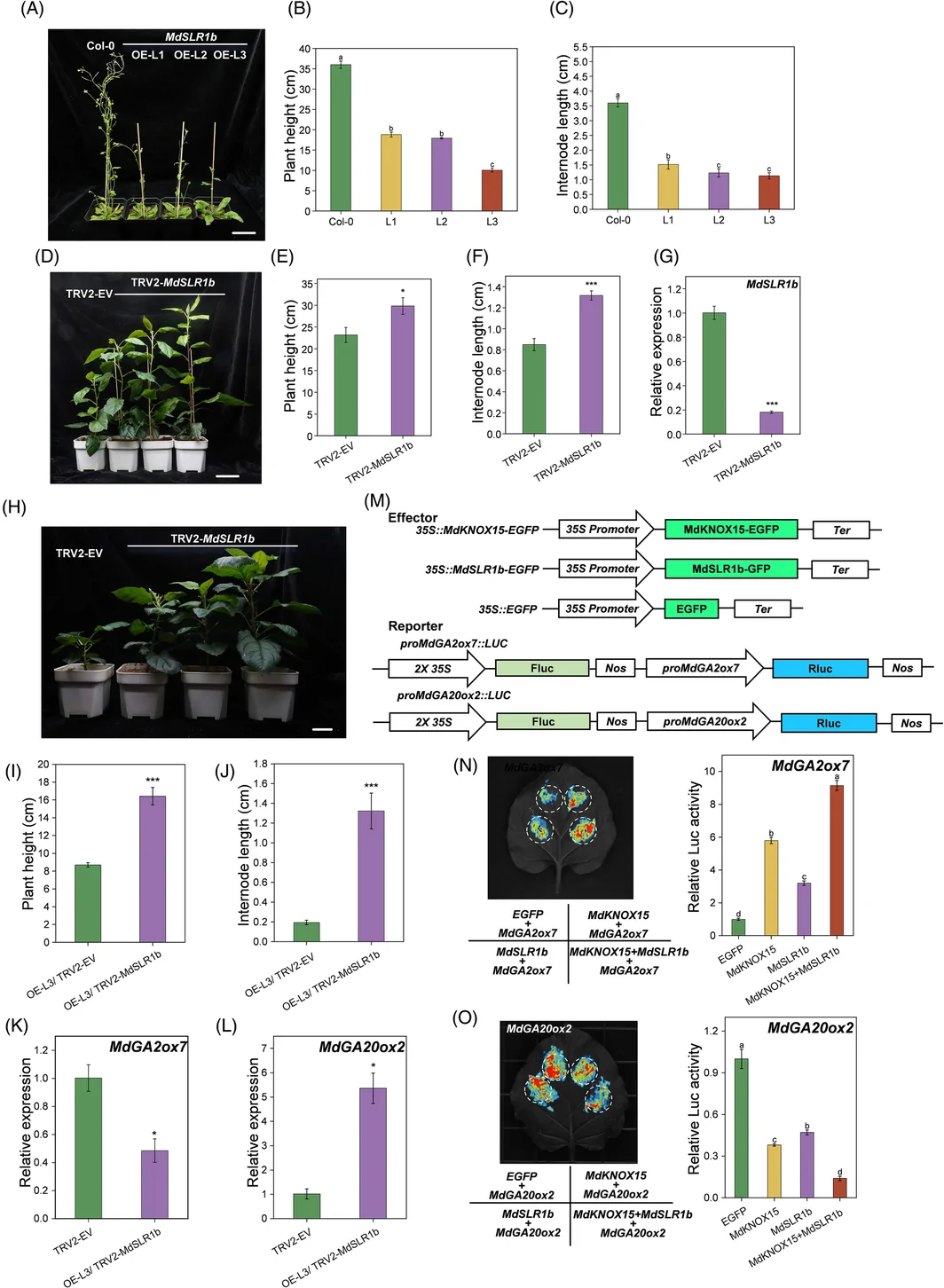
MdKNOX15 and MdSLR1b synergistically regulate plant height and target genes. (A–G) MdSLR1b negatively regulates plant height. (A–C) *MdSLR1b* overexpression (*MdSLR1b*‐OE) causes dwarfing in transgenic Arabidopsis. (A) Plant photographs. (B) Plant height. (C) Internode length. (D–G) VIGS‐mediated knockdown of *MdSLR1b* promotes apple plant elongation. (D) Photographs of 2‐month‐old *MdSLR1b* knockdown apple plants. (E) Relative expression level of *MdSLR1b* in knockdown plants, with empty vector control. (F) Plant height. (G) Internode length. (H–O) MdKNOX15 and MdSLR1b synergistically regulate apple plant height and downstream gene. (H–J) Phenotypic analysis of *MdSLR1b* knockdown in the *MdKNOX15*‐OE (L3) background. (H) Photographs of 2‐month‐old *MdSLR1b* knockdown apple plants. (I) Plant height. (J) Internode length. (K, L) Expression levels of *MdGA2ox7* (K) and *MdGA20ox2* (L) after *MdSLR1b* knockdown in the *MdKNOX15*‐OE (L3) background. Empty vector was used as control in (H–L). (M–O) Effect of MdKNOX15 and MdSLR1b on the promoter activities of *MdGA2ox7* and *MdGA20ox2*. (M) Schematic diagram of effector and reporter constructs. (N) Luciferase signal and ratio showing the effect on the *MdGA2ox7* promoter. (O) Luciferase signal and ratio showing the effect on the *MdGA20ox2* promoter. Scale bars = 5 cm. Statistical significance was determined using Student's *t*‐test for comparisons between two groups, and asterisks (*) above the bar charts indicate significant differences between TRV‐EV and TRV2‐*MdSLR1b* plants (*P < 0.05; **P < 0.01; ***P < 0.001). One‐way analysis of variance (ANOVA) followed by Tukey's test was used for multiple comparisons, and different lowercase letters above the bar charts indicate significant differences between distinct plants (B, C) or effectors (N, O) (*P* < 0.05).

To verify whether MdSLR1b regulates plant height, transgenic Arabidopsis plants overexpressing *MdSLR1b* were generated. The results showed that the *MdSLR1b*‐OE lines exhibited a pronounced dwarf phenotype (Figure 6A–C). In addition, transgenic apple lines with *MdSLR1b* knockdown were constructed using VIGS technology. When the expression level of *MdSLR1b* was suppressed, the plant height of the transgenic apple lines was significantly increased (Figure 6D–G). These results indicate that MdSLR1b negatively regulates plant height.

### MdKNOX15 and MdSLR1b cooperatively regulate plant height and gene expression

To confirm the combined role of *MdSLR1b* and *MdKNOX15* in regulating plant height, knockdown of *MdSLR1b* was performed in *MdKNOX15*‐OE transgenic apple plants. Phenotypic analysis revealed that plant height and internode length were significantly increased in these lines compared with the empty vector control (Figure 6H–J). Meanwhile, gene expression analysis showed that when *MdSLR1b* was knocked down, the expression level of *MdGA2ox7* was significantly reduced, whereas that of *MdGA20ox2* was markedly elevated. Therefore, MdSLR1b and MdKNOX15 may synergistically regulate the expression of *MdGA2ox7* and *MdGA20ox2* (Figure 6K,L). A dual‐luciferase reporter assay was further employed to investigate the effects of MdSLR1b and MdKNOX15 on the promoter activities of downstream genes (Figure 6M). The results showed that the introduction of MdSLR1b further amplified the activation of the *MdGA2ox7* promoter by MdKNOX15 (Figure 6N) while also enhancing the inhibitory effect of MdKNOX15 on the *MdGA2ox7* promoter (Figure 6O). Based on the above data, it is concluded that MdKNOX15 and MdSLR1b synergistically regulate plant height and gene expression levels.

## DISCUSSION

GAs are central hormones regulating stem elongation, and the balance between their biosynthesis and catabolism determines plant height. In this study, we found that *MdKNOX15* is highly expressed in spur‐type apple varieties and dwarfing rootstocks (Figure 1). A single‐nucleotide polymorphism (SNP) within the promoter may affect the recognition and binding of its upstream regulatory factors, leading to significant changes in expression levels (Zhang et al., 2018). This differential expression prompted us to examine the promoter sequences of *MdKNOX15* in different germplasms. Cloning and sequencing of the 1.5‐kb regulatory region upstream of the start codon revealed multiple SNPs and InDels; however, these did not show a consistent distribution between dwarf and standard types (Figure S9a). Additionally, analysis of available genome sequencing data did not identify any potentially functional SNPs or InDels (Figure S9b) within the 2.0‐kb promoter region of *MdKNOX15*. Therefore, whether the differential expression of *MdKNOX15* is caused by promoter variation or differences in the abundance of upstream regulators warrants further investigation. *MdKNOX15* overexpression led to a significant decrease in endogenous bioactive GA content, a typical dwarf phenotype, and shortened internode cell length. Conversely, silencing *MdKNOX15* promoted GA accumulation and increased plant height (Figure 2). These results indicate that MdKNOX15 participates in apple height regulation by negatively regulating GA levels.

The classic Green Revolution genes, such as rice *sd1* (encoding GA20ox), represent the dwarfing mechanism caused by GA deficiency (Sasaki et al., 2002). Broader studies have shown that both GA biosynthesis and metabolism affect bioactive GA content, thereby modulating plant height. Among them, *AtGA20ox* promotes hypocotyl and internode elongation (Rieu et al., 2008), whereas *AtGA2ox1* significantly reduces bioactive GA levels and improves field performance in turfgrass (Agharkar et al., 2007). By integrating DAP‐seq and RNA‐seq data, we identified two key GA synthesis/metabolism‐related genes *MdGA2ox7* (GA metabolism) and *MdGA20ox2* (GA synthesis) which are directly targeted by MdKNOX15 (Figure 4). RT‐qPCR, EMSA, Y1H, ChIP‐qPCR, and dual‐luciferase reporter assays confirmed that MdKNOX15 binds to the promoters of these two genes, activating *MdGA2ox7* expression and repressing *MdGA20ox2* expression, thereby bidirectionally reducing bioactive GA levels. We previously showed that heterologous overexpression of the MdKNOX15 target gene *MdGA2ox7* reduces GA content and causes dwarfism in Arabidopsis (Yan et al., 2024); here, *MdGA2ox7* knockdown increased GA content and apple plant height (Figure S6). Meanwhile, *MdGA20ox2* heterologous overexpression increased plant height in Arabidopsis, whereas *MdGA20ox2* knockdown caused reduced GA content and dwarfism in apple (Figure S7). Alternative splicing of *PeGA20ox1* in Moso bamboo leads to loss of function, disturbs GA homeostasis, and inhibits internode elongation (Di et al., 2026). Although the function of KNOX transcription factors in maintaining low GA levels to inhibit differentiation has been reported in herbaceous plants such as Arabidopsis and rice (Hay et al., 2002; Tsuda et al., 2011), this study is the first to reveal the molecular mechanism by which a KNOX transcription factor suppresses GA accumulation through dual regulation of GA biosynthetic and catabolic enzyme genes in apple, providing a new target for dwarf breeding in woody crops.

In this study, we found that exogenous GA treatment could not fully restore the plant height phenotype of *MdKNOX15* overexpression lines (Figure 3), prompting us to investigate whether MdKNOX15 also affects GA signaling. Wheat *Rht*, which encodes a DELLA protein, represents another dwarfing mechanism, namely, GA signal insensitivity (Peng et al., 1999). DELLA proteins are core repressors in the GA signaling pathway, and their stability is regulated by the ubiquitin–26S proteasome pathway (Alabadí & Sun, 2025). Through yeast two‐hybrid screening, we obtained the DELLA protein homolog MdSLR1b (homologous to rice SLR1) and confirmed that it directly interacts with MdKNOX15 in the nucleus (Figure 5). Further investigation showed that *MdSLR1b* overexpression inhibits plant height, whereas *MdSLR1b* silencing alleviates the dwarf phenotype of *MdKNOX15* overexpression lines (Figure 6). In tomato, CRISPR editing of DELLA‐encoding genes created dominant dwarf alleles that are resistant to degradation (Tomlinson et al., 2019), demonstrating that stabilization of DELLA proteins is an effective strategy for generating dwarf phenotypes. Meanwhile, Mediator, as a transcriptional co‐activator, activates GA signaling by promoting DELLA protein degradation (Ohama et al., 2025). Protein stability assays showed that MdKNOX15 significantly delayed the degradation of MdSLR1b, and especially in the presence of GA, MdSLR1b protein levels were maintained (Figure 5). This suggests that MdKNOX15 enhances MdSLR1b protein stability through protein–protein interaction, thereby strengthening its repression of GA signaling. This mechanism differs from the classic DELLA degradation pathway and reveals a transcription factor‐mediated mode of DELLA stabilization via post‐translational regulation, providing a new perspective for understanding non‐classical DELLA regulation in plant height.

The transcriptional regulation of downstream genes by KNOX transcription factors can be influenced by their interactions with other proteins. For example, in lettuce, the LsKANT3‐LsBLH2‐LsOFP6 complex regulates bolting by coordinately modulating GA synthesis and catabolism genes (Chen et al., 2025). After clarifying that MdKNOX15 simultaneously regulates GA metabolic enzyme genes and DELLA protein stability, we further explored whether they act synergistically. Silencing *MdSLR1b* in the *MdKNOX15*‐OE background significantly inhibited *MdGA2ox7* expression and restored *MdGA20ox2* expression, while partially reversing the dwarf phenotype (Figure 6). This indicates that the two factors may synergistically regulate GA metabolism genes. Similarly, the wheat Green Revolution gene *Rht8* interacts with TaEIL1 to form condensates that affect the expression of the downstream GA synthesis gene *TaGA3ox2*, thereby fine‐tuning plant height and internode elongation (Dong et al., 2026). It is possible that the MdKNOX15‐MdSLR1b complex might jointly bind to the promoters of *MdGA2ox7* and *MdGA20ox2* to potentially coordinate their transcription. However, whether MdSLR1b directly binds DNA remains unclear, because DELLAs lack a canonical DNA‐binding motif (Fukazawa et al., 2017). Previous studies have suggested that DELLA proteins act as transcriptional co‐regulators. They interact with various transcription factors to modulate target gene expression (Fukazawa et al., 2021; Ma et al., 2026). Meanwhile, DELLAs have been demonstrated to be precisely involved in three‐dimensional chromatin structure and in regulating the balance between plant growth and metabolism (Li et al., 2018; Liu et al., 2025; Wu et al., 2020). This is highly consistent with the functional logic of the MdKNOX15‐MdSLR1b module in this study, in which MdKNOX15 and MdSLR1b synergistically activate the promoters of two downstream genes. Moreover, knockout of the rice DELLA‐encoding gene *SLR1* or the wheat Green Revolution allele *Rht‐B1b* significantly reduces salt tolerance (Cheng et al., 2026), whereas overexpression of WT *SLR1* or DELLA proteins significantly enhances salt tolerance in rice or *Malus sieversii* (Wang et al., 2026), indicating that DELLA positively regulates plant salt tolerance. Considering that the downstream target gene *MdGA2ox7* and its homolog *AtGA2ox7* positively regulate salt tolerance (Magome et al., 2008; Yan et al., 2024), and that fine‐tuning GA improves alkali‐heat tolerance (Guo et al., 2025), whether MdKNOX15 and MdSLR1b are involved in the response to saline–alkali stress in apple presents an interesting direction for future research.

**Figure 7:**
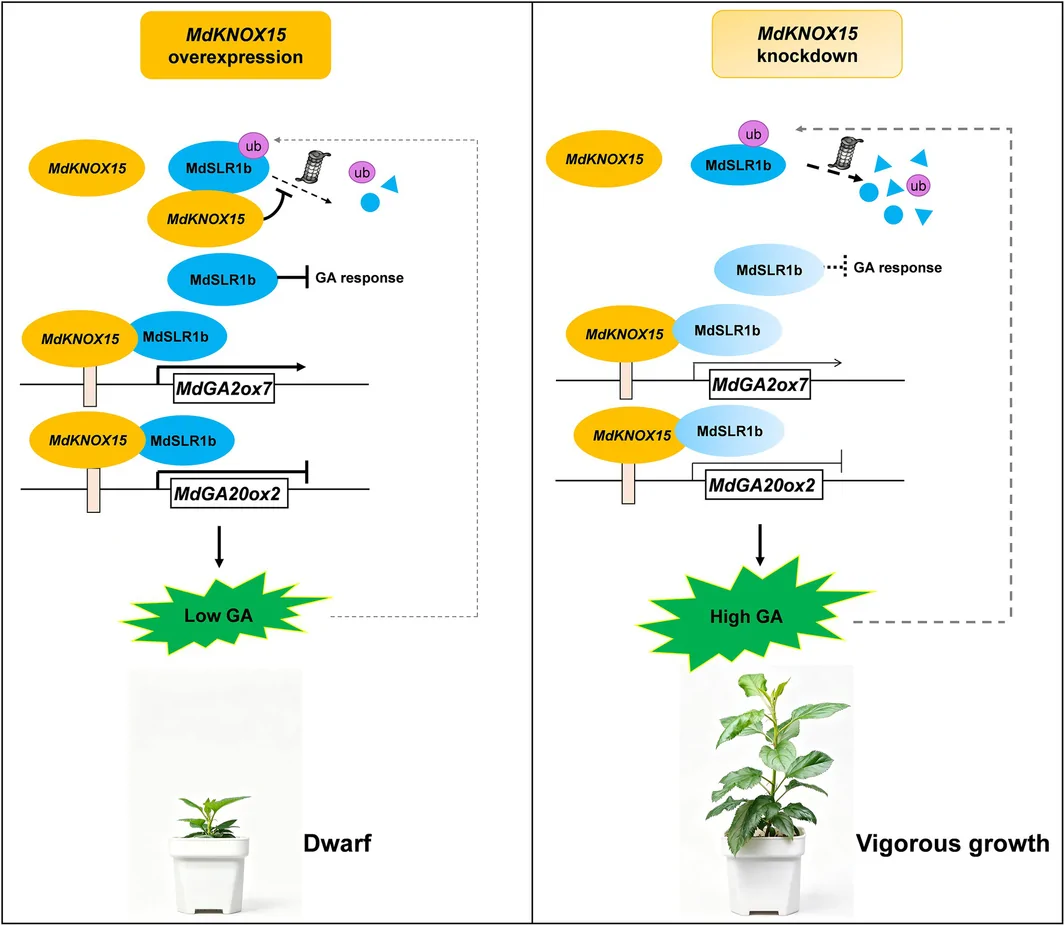
Proposed model for MdKNOX15‐mediated regulation of apple plant height. In *MdKNOX15* overexpression plants, MdKNOX15 directly represses *MdGA20ox2* and activates *MdGA2ox7*, reducing bioactive GA levels. At the same time, it interacts with the DELLA protein MdSLR1b, preventing MdSLR1b degradation to enhance the suppression of GA response signaling. The reduction in GA content and the enhanced inhibition of GA signaling synergistically block stem cell elongation, resulting in a dwarf phenotype. Conversely, in *MdKNOX15* knockdown plants, MdSLR1b degradation is accelerated, weakening the inhibition of GA signaling. In addition, the transcriptional regulatory activity of the MdKNOX15‐MdSLR1b complex on *MdGA20ox7*/*MdGA20ox2* is reduced, GA content increases, and plants exhibit vigorous growth.

In summary, we propose a “dual brake” model centered on MdKNOX15. On the one hand, MdKNOX15 directly represses GA biosynthesis genes and activates GA catabolism genes, thereby reducing bioactive GA levels. On the other hand, MdKNOX15 interacts with the DELLA protein MdSLR1b to delay its degradation, thereby enhancing the repression of the GA signaling pathway. Together, these two mechanisms synergistically inhibit stem elongation, leading to the dwarf phenotype in apple (Figure 7). This module not only theoretically enriches the molecular network regulating plant height in woody plants but also provides actionable gene editing targets for dwarf breeding of perennial fruit trees such as apple, offering significant application potential.

## MATERIALS AND METHODS

### Plant materials and growth conditions

Six standard‐type apple varieties (“Pacific Gala,” “Changhong,” “Jiping 1,” “Luli,” “Wanglin,” “Guohong”) and four spur‐type apple varieties (“Shifu Spur,” “Miyazaki Spur,” “Tianhong 2,” “Jinzhouhong”) were planted at the Shijiazhuang Institute of Fruit Tree Research, Hebei Academy of Agriculture and Forestry Sciences (Shijiazhuang, Hebei, China; 38.1222°N, 114.52046°E). Spring shoot tips were collected, immediately frozen in liquid nitrogen, and stored at −80°C until use. Two‐month‐old potted seedlings of dwarfing (M9T337, M26) and standard rootstocks (*Malus micromalus* and *Malus prunifolia*) were grown in a greenhouse under a 16 h light/8 h dark photoperiod at 23°C. Shoot tips were collected, snap‐frozen in liquid nitrogen, and stored at −80°C.

Apple tissue‐cultured or potted seedlings (*Malus × domestica* “GL‐3”) were cultured at 23°C under a 16 h light/8 h dark photoperiod. *Arabidopsis thaliana* (Col‐0) and *Nicotiana benthamiana* were grown under the same conditions.

### Gene cloning and vector construction

The coding sequences of full‐length *MdKNOX15*, *MdGA2ox7*, *MdGA20ox2*, and *MdSLR1b* were amplified from cDNA of apple shoot tips (*Malus domestica* cv. “Golden Delicious”) and used to construct plant overexpression vectors pCAMBIA2300 or pCAMBIA1301. Specific fragments of *MdKNOX15*, *MdGA2ox7*, *MdGA20ox2*, and *MdSLR1b* were cloned into the pTRV2 vector to generate silencing constructs. To clone the promoter sequences of *MdKNOX15* from different germplasm materials, genomic DNA was extracted from leaves of various apple varieties and used as a template for PCR amplification. The amplified products were ligated into the cloning vector (pBM16K Toposmart Cloning Kit, Biomed, China) and transformed into *Escherichia coli* for sequencing. Primers used for gene cloning and plasmid construction are listed in Table S1.

### Genetic transformation, screening, and identification

Overexpression transgenic lines of apple were generated according to a previous method (Zhang et al., 2024). Briefly, leaves from 1‐month‐old “GL‐3” apple tissue‐cultured seedlings were wounded with a sterile scalpel and immersed in *Agrobacterium* infection solution for 10 min. Infected leaves were kept in the dark for 3 days and then transferred to light conditions (16 h light/8 h dark) at 24°C until new shoots emerged from the wound sites. Regenerated plants with kanamycin resistance were further confirmed by PCR and Western blotting.

VIGS was used to generate knockdown plants of the corresponding genes in *M. hupehensis* (Pingyi Tiancha) and “GL‐3.” For *M. hupehensis*, the published method was followed (Zeng et al., 2021). Briefly, seeds that had been stratified and germinated to approximately 0.5 cm were immersed in bacterial solution and subjected to a vacuum of −25 kPa for about 10 sec, followed by rapid release to atmospheric pressure. This operation was repeated twice. After vacuum infiltration, the germinated seeds were dried on filter paper, incubated in the dark for 3 days, rinsed with clean water, and sown in nutrient pots. For “GL‐3,” the reported method was used (Zhang et al., 2024). Briefly, “GL‐3” plantlets with complete root systems after 1 month of rooting culture were immersed in *Agrobacterium* solution containing TRV1 and TRV2 fusion vectors, treated under a vacuum of −60 kPa for about 30 min, transferred to nutrient pots, kept in the dark for 16 h, and then cultured under normal conditions.

Transgenic Arabidopsis plants were generated by the floral dip method as previously described (Zhang et al., 2006).

### Hormone measurement and treatments

Leaves of transgenic apple plants and shoot tips of different varieties were collected for hormone measurement. Approximately, 0.5 g of freeze‐dried sample was extracted with 3 mL of methanol/water/formic acid (15:4:1 v/v/v) and incubated at 4°C in the dark for 16 h with thorough mixing. After low‐temperature ultrasonication for 30 min, the mixture was centrifuged at 12000 rpm for 5 min at 4°C. The supernatant was collected and filtered through a 0.22 μm organic phase membrane prior to high‐performance liquid chromatography (HPLC)‐MS/MS analysis. HPLC coupled to an Agilent 6495C triple quadrupole mass spectrometer was used for analysis. The chromatographic conditions were set as follows: column, Poroshell SB‐C18 reversed‐phase column (2.1 × 50 mm, 2.7 μm); column temperature, 40°C; mobile phase, 0.05% formic acid in water and acetonitrile with gradient elution; injection volume, 0.5 μL. The mass spectrometric parameters were set as follows: ionization mode, positive and negative electrospray ionization (ESI) modes; scan type, multiple reaction monitoring (MRM); gas temperature, 200°C; gas flow, 14 L/min; nebulizer, 35 psi; sheath gas temperature, 300°C; sheath gas flow, 11 L/min; capillary voltages, +4000 V and − 3500 V; nozzle voltages, +1500 V and − 1500 V. Standards were purchased from Sigma‐Aldrich (GA_3_, G7645; GA_4_, G7276) and Yuanye Bio‐Technology Co., Ltd. (IAA, B21810; BR, B21633; Zeatin, B25664). Three biological replicates were used for each hormone measurement.

For growth regulator spray treatments, WT “GL‐3” and *MdKNOX15* overexpression apple plants were sprayed with different concentrations (0, 1, 5, 10 mM) of exogenous GA_3_. The treatment was applied twice every 10 days, and plant height and internode length were measured after 1 month. Meanwhile, 100 μM UCZ was sprayed on WT and transgenic apple plants twice a week for 1 month, after which plant height and internode length were recorded.

### RNA‐Seq and DAP‐seq

Shoot tips were randomly collected from 2‐month‐old wild‐type (WT, “GL‐3”) and *MdKNOX15*‐OE apple plants. RNA‐seq library construction and sequencing were performed by Shanghai Meiji Biotechnology Co., Ltd. on the DNBSEQ‐T7RS platform (PE150). Raw sequence data were deposited in the NCBI SRA database under accession number PRJNA1444345. Bioinformatics analysis was carried out using the Majorbio Cloud platform (https://cloud.majorbio.com). Briefly, clean reads were aligned to the reference genome using HISAT2 (v2.1.0), and transcript abundance was calculated as FPKM. Differentially expressed genes were identified with |log_2_FC| ≥ 1 and FDR ≤0.05. KEGG enrichment analyses were conducted using the clusterProfiler package.

For DAP‐seq, genomic DNA was extracted from “Golden Delicious” apple using standard protocols. DNA was fragmented to ~200 bp with a Covaris M220 ultrasonicator. Libraries were prepared with the NEXTflex Rapid DNA‐Seq Kit 2.0 per the manufacturer's instructions. Purified Halo‐MdKNOX15 fusion was mixed with the library for protein–DNA binding. After washing, bound DNA was eluted, amplified with indexed primers, and sequenced. Raw data were deposited in NCBI (PRJNA1450900) and aligned to the reference apple genome. Peaks were called using MACS2 (‐‐fe‐cutoff 2 ‐q 0.05) to identify significant binding sites.

### Yeast two‐hybrid (Y2H)

Y2H screening was performed using the Matchmaker Gold Y2H System (Clontech) as previously described (Jia, Xing, Zhang, Chen, et al., 2021). A cDNA library constructed by TaKaRa Company from apple buds was screened using MdKNOX15 as the bait. For point‐to‐point validation, the pGBKT7‐MdKNOX15 and the pGADT7‐MdSLR1b plasmids were transformed into the Y2HGold yeast and then cultured on SD/−T‐L and SD/−T‐L‐A‐H medium.

### Pull‐down, LCA, and BiFC

For GST pull‐down assay, the GST‐MdKNOX15 and MdSLR1b‐HIS recombinant proteins were prepared using pGEX4t‐1 and pET28a vectors, respectively, and expressed in BL21(DE3)pLysS *E. coli* host cells upon induction with 0.5 mM IPTG. The GST‐MdKNOX15 and MdSLR1b‐HIS fusion protein was then bound to glutathione Sepharose beads, and the pull‐down was performed according to the manufacturer's instructions (Thermo Fisher Scientific, USA). After extensive washing, the bound proteins were eluted and detected by Western blotting using anti‐HIS (1B7G5, proteintech, China) and anti‐GST antibodies (3G12B10, proteintech, China), respectively, as described previously (Jia, Xing, Zhang, Chen, et al., 2021).

For LCA, cLUC‐MdKNOX15 and nLUC‐MdSLR1b plant expression vectors were constructed by fusing the coding sequence (CDS) of MdKNOX15 to the C‐terminal half of LUC and the CDS of MdSLR1b to the N‐terminal half of LUC. The recombinant vectors were co‐infiltrated into tobacco leaf. Luciferase activity signals were then detected using a plant *in vivo* imaging system (Zhang et al., 2022).

For bimolecular fluorescence complementation (BiFC) assays, plant expression vectors encoding MdKNOX15‐YFP^NE^ and MdSLR1b‐YFP^CE^ were constructed using the pSPYNE and pSPYCE plasmids (Walter et al., 2004), respectively. The recombinant vectors were co‐infiltrated into tobacco leaf via *Agrobacterium*, and the YFP signals were detected.

### Electrophoretic mobility shift assay (EMSA)

The His‐tagged MdKNOX15 protein was induced as described in Ssection 4.7 and purified using a nickel ion column (Ni‐NTA, Millipore, USA). The purified MdKNOX15‐HIS fusion protein was then incubated with various probes. Protein–probe complexes were resolved by electrophoresis and detected using the LightShift Chemiluminescent EMSA Kit (Thermo Fisher Scientific, USA).

### Yeast one‐hybrid and ChIP‐qPCR assays

For Y1H, the promoter fragments of *MdGA2ox7* and *MdGA20ox2* were cloned into the pHIS2 vector as bait, and the CDS of *MdKNOX15* was cloned into the pGADT7 vector as prey. The two constructs were co‐transformed into Y187 yeast cells and grown on medium containing 3‐AT.

ChIP‐qPCR assays were performed as described previously (Mao et al., 2023). Briefly, protein–DNA complexes were cross‐linked in MdKNOX15‐FLAG transgenic apple (OE‐L3) shoots using formaldehyde. After sonication to shear the chromatin, the protein–DNA complexes were immunoprecipitated with an anti‐FLAG antibody (F1804, Sigma‐Aldrich, USA). The enriched DNA fragments were analyzed by quantitative real‐time PCR using the specific primers listed in Table S2. *MdMDH* gene and no antibody served as the negative controls in ChIP‐qPCR.

### Dual‐luciferase reporter assay

For the dual‐luciferase reporter assay, the promoters of *MdGA2ox7* and *MdGA20ox2* were separately cloned into the pCAMBIA0390‐Dluc vector as reporters, while the CDS of *MdKNOX15* and *MdSLR1b* were respectively inserted into the pCAMBIA2300 vector to generate effectors. These two constructs were co‐infiltrated into tobacco leaves via *Agrobacterium*. Luminescence signals were then measured, and the luciferase activity ratio was calculated according to a previously established method (Jia, Xing, Zhang, Zhang, et al., 2021).

### Cell‐free protein degradation assay

Purified MdSLR1b‐HIS protein was allowed to incubate with the crude protein extracted from *MdKNOX15*‐OE or WT “GL‐3” leaves, and samples were harvested at multiple time intervals. For 26S proteasome inhibitor treatments, 50 μM MG132 was added before incubation. Protein abundance was assessed by Western blotting.

### Degradation assays in tobacco

An *Agrobacterium tumefaciens* infiltration solution containing the constructs CaMV35S::*MdKNOX15*‐FLAG and CaMV35S::*MdSLR1b*‐HA was prepared by mixing equal volumes of bacterial cultures carrying each recombinant vector. This solution was then infiltrated into tobacco leaves. After 3 days of normal cultivation, proteins were extracted. For MG132 treatment, leaves were infiltrated with a 25 μM solution of the inhibitor 16 h prior to sampling. For GA or UCZ treatment, 5 mM GA_3_ or 100 μM UCZ was applied by spraying 24 h prior to sampling. Finally, the abundance of MdSLR1b‐HA proteins was analyzed by Western blotting.

### RT‐qPCR

Total RNA was isolated using the RNA extraction kit (OMEGA, USA), followed by reverse transcription. RT‐qPCR analyses were conducted on the ABI Prism 7500 system, using *MdActin* as the internal control. Primers employed for RT‐qPCR are detailed in Table S2.

### Data analysis

All data were analyzed using IBM SPSS Statistics 26 and graphed with Origin 2024 software. Data are presented as mean ± standard deviation (SD) from three independent biological replicates. For comparisons between two groups, Student's *t*‐test was applied, and significance was indicated as *P < 0.05, **P < 0.01, and ***P < 0.001. For multiple comparisons, one‐way analysis of variance (ANOVA) was performed followed by Tukey's multiple comparison test, and different uppercase/lowercase letters indicate significant differences at *P* < 0.05.

## Author Contributions

PJ, GQ, and XZ conceived and designed the experiments. RY, XZ, YW, YY, BL, and XZ performed the experiments and collected the data. RY, NW, and GQ analyzed the data. RY drafted the manuscript. GQ, YW, and PJ revised and finalized the manuscript. All authors read and approved the final manuscript.

## Conflicts of Interest

The authors declare no conflicts of interest.

## Supporting information


**Figure S1.** Spring shoots of standard‐type and spur‐type apple varieties. (a)–(j) Photographs of typical spring shoots from each variety. (a) “Pacific Gala,” TPY‐GL; (b) “Changhong,” ChangH; (c) “Jiping 1,” JiP1; (d) “Luli”; (e) “Wanglin,” WangL; (f) “Guohong,” GuoH; (g) “Shifu Spur,” SF; (h) “Miyazaki Spur,” Mz; (i) “Tianhong 2,” TianH2; (j) “Jinzhouhong,” JinZH. Scale bars represent 5 cm. (k) Statistical analysis of branch length. Data are mean ± SD (*n* = 10 branches). The significance test was carried out by one‐way analysis of variance (ANOVA) followed by Tukey's test. Different lowercase letters above the bars indicate significant differences among branches of different apple varieties (*P* < 0.05).
**Figure S2.** Identification of transgenic apple plants. (a–d) Identification of *MdKNOX15* overexpression lines. (a) PCR detection using genomic DNA as template. (b) RT‐PCR detection using total RNA as template. For (a) and (b), PCR primers were gene‐specific forward primer for *MdKNOX15* and reverse primer for the FLAG tag. (c) Western blot analysis of total protein using anti‐FLAG antibody to detect MdKNOX15‐FLAG; Actin served as a loading control. (d) Relative expression level of *MdKNOX15* measured by RT‐qPCR. “GL‐3” was used as the wild‐type control. (e) *MdKNOX15* expression analysis in *MdKNOX15* knockdown transgenic “GL‐3” apple. (f) *MdKNOX15* expression analysis in *MdKNOX15* knockdown transgenic *M. hupehensis* apple. For (e) and (f), plants transformed with TRV2 empty vector served as controls. Data are mean ± SD of three independent experiments. Statistical significance was determined using Student's *t*‐test for comparisons between TRV2‐EV and TRV2‐*MdKNOX15* plants, and asterisks (*) above the bar charts indicate significant differences (*P < 0.05; **P < 0.01; ***P < 0.001). One‐way analysis of variance (ANOVA) followed by Tukey's test was used for multiple comparisons, and different lowercase letters above the bar charts indicate significant differences among WT and various *MdKNOX15*‐OE transgenic plants (*P* < 0.05).
**Figure S3.** Determination of endogenous phytohormone contents in *MdKNOX15* transgenic plants. (a) Auxin (3‐Indoleacetic acid, IAA); (b) Brassinosteroids (BR); (c) Cytokinins (Zeatin). Data are mean ± SD of three independent experiments. One‐way analysis of variance (ANOVA) followed by Tukey's test was used for multiple comparisons, and different lowercase letters above the bar charts indicate significant differences among WT and various *MdKNOX15*‐OE transgenic plants (*P* < 0.05).
**Figure S4.** Number of differentially expressed genes identified by RNA‐seq between the *MdKNOX15*‐OE line and WT.
**Figure S5.** Expression analysis of *MdGA2ox7* and *MdGA20ox2* in different apple varieties. (a, b) Expression levels of *MdGA2ox7* (a) and *MdGA20ox2* (b) in standard‐type and spur‐type varieties. (c, d) Expression levels of *MdGA2ox7* (c) and *MdGA20ox2* (d) in dwarfing and standard rootstocks. Data are means ± SD of three independent experiments. The significance test was carried out by one‐way analysis of variance (ANOVA) followed by Tukey's test, and different lowercase letters above the bars indicate significant differences among branches of different apple varieties (*P* < 0.05).
**Figure S6.** Effects of *MdGA2ox7* on apple plant height and GA content. (a) Phenotypic photograph of *MdGA2ox7* knockdown transgenic apple plants. (b) Relative expression level of *MdGA2ox7*. (c) Plant height. (d) Internode length. Scale bar = 5 cm. (e) GA_3_ content. (f) GA_4_ content. Statistical significance was determined using Student's *t*‐test for comparisons between TRV2‐EV and TRV2‐*MdGA2ox7* plants, and asterisks (*) above the bar charts indicate significant differences (*P < 0.05; **P < 0.01; ***P < 0.001).
**Figure S7.** Effects of *MdGA20ox2* on plant height and GA content. (a) Subcellular localization of MdGA20ox2. Scale bar = 20 μm. (b–d) *MdGA20ox2* overexpression in transgenic Arabidopsis. (b) Plant photographs. (c) Plant height. (d) Internode length. In (c) and (d), one‐way analysis of variance (ANOVA) followed by Tukey's test was used for multiple comparisons, and different lowercase letters above the bar charts indicate significant differences among Col‐0 and various *MdGA20ox2*‐OE transgenic plants (*P* < 0.05). (e–j) Effects of *MdGA20ox2* knockdown in apple on plant height and GA content. (e) Plant photographs. (f) Relative expression level of *MdGA20ox2*. (g) Plant height. (h) Internode length. (i) GA_3_ content. (j) GA_4_ content. In figures (f), (g), (h), (i), and (j), statistical significance was determined using Student's *t*‐test for comparisons between TRV2‐EV and TRV2‐*MdGA20ox2* plants, and asterisks (*) above the bar charts indicate significant differences (*P < 0.05; **P < 0.01; ***P < 0.001). (k–m) Effect of exogenous GA_3_ treatment on *MdGA20ox2* knockdown apple. (k) plant photographs. (l) plant height. (m) internode length. For (b), (e), and (k), scale bars = 5 cm. In figures (l) and (m), asterisks (*) above the bars indicate significant differences between different treatments within the same line (*P < 0.05; **P < 0.01; ***P < 0.001); different uppercase and lowercase letters above the bars represented significant differences among different lines under the same treatment condition at the *P* < 0.05 level.
**Figure S8.** Sequence alignment of MdSLR1b with rice SLR1.
**Figure S9.** Alignment analysis of *MdKNOX15* promoter sequences in different apple germplasms. (a) Sequence alignment of the promoter region upstream of the *MdKNOX15* start codon obtained by PCR cloning. (b) Sequence alignment of the promoter region upstream of the *MdKNOX15* start codon from some published apple genomes.
**Table S1.** Primers used for vector construction.
**Table S2.** Primers used for RT‐qPCR and ChIP‐qPCR.

## Data Availability

All data supporting the findings of this study are available within the manuscript and its Supporting Information. The RNA‐Seq and DAP‐seq datasets generated in this study are available from the Sequence Read Archive (SRA) under accession number PRJNA1444345 (https://dataview.ncbi.nlm.nih.gov/object/PRJNA1444345?reviewer=2q0cdlu0u827lvti0jqh9i7iif) and PRJNA1450900 (https://dataview.ncbi.nlm.nih.gov/object/PRJNA1450900?reviewer=8aeo8pbh5p446aehnh5obabg3f), respectively.
