# Supplementary material for: The MdSLR1b‐MdKNOX15‐MdGA20ox2/MdGA2ox7 module regulates dwarfing by mediating the gibberellin pathway in apple
Source: Plant J. 2026 Sep 19;127(6):e71137. doi: 10.1111/tpj.71137 (PMC13589610; doi:10.1111/tpj.71137)
Supplement: Supplementary file 1 [file TPJ-127-0-s001.docx]

**Supporting Information**

**FIGURE S1.** **Spring shoots of standard-type and spur‑type apple varieties.** (a)-(j) Photographs of typical spring shoots from each variety. (a) ‘Pacific Gala’, TPY-GL; (b) ‘Changhong’, ChangH; (c) ‘Jiping 1’, JiP1; (d) ‘Luli’; (e) ‘Wanglin’, WangL; (f) ‘Guohong’, GuoH; (g) ‘Shifu Spur’, SF; (h) ‘Miyazaki Spur’, Mz; (i) ‘Tianhong 2’, TianH2; (j) ‘Jinzhouhong’, JinZH. Scale bars represent 5 cm. (k) Statistical analysis of branch length. Data are means ± SD (n= 10 branches). The significance test was carried out by one-way analysis of variance (ANOVA) followed by Tukey’s test. Different lowercase letters above the bars indicate significant differences among branches of different apple varieties (*p* < 0.05).

**FIGURE S2. Identification of transgenic apple plants.** (a‑d) Identification of *MdKNOX15* overexpression lines. (a) PCR detection using genomic DNA as template. (b) RT‑PCR detection using total RNA as template. For (a) and (b), PCR primers were gene‑specific forward primer for *MdKNOX15* and reverse primer for the FLAG tag. (c) Western blot analysis of total protein using anti‑FLAG antibody to detect MdKNOX15‑FLAG; Actin served as a loading control. (d) Relative expression level of *MdKNOX15* measured by RT‑qPCR. ‘GL-3’ was used as the wild‑type control. (e) *MdKNOX15* expression analysis in *MdKNOX15* knockdown transgenic ‘GL-3’ apple. (f) *MdKNOX15* expression analysis in *MdKNOX15* knockdown transgenic *M. hupehensis* apple. For (e) and (f), plants transformed with TRV2 empty vector served as controls. Data are means ± SD of three independent experiments. Statistical significance was determined using Student’s *t*-test for comparisons between TRV2-EV and TRV2-*MdKNOX15* plants, and asterisks (*) above the bar charts indicate significant differences (**p* < 0.05; ***p* < 0.01; ****p* < 0.001). One-way analysis of variance (ANOVA) followed by Tukey’s test was used for multiple comparisons, and different lowercase letters above the bar charts indicate significant differences among WT and various *MdKNOX15*-OE transgenic plants (*p* < 0.05).

**FIGURE S3. Determination of endogenous phytohormone contents in *MdKNOX15* transgenic plants.** (a) Auxin (3-Indoleacetic acid, IAA); (b) Brassinosteroids (BR); (c) Cytokinins (Zeatin). Data are means ± SD of three independent experiments. One-way analysis of variance (ANOVA) followed by Tukey’s test was used for multiple comparisons, and different lowercase letters above the bar charts indicate significant differences among WT and various *MdKNOX15*-OE transgenic plants (*p* < 0.05).

**FIGURE S4.** **Number of differentially expressed genes identified by RNA‑seq between the *MdKNOX15*‑OE line and WT.**

**Figure S5. Expression analysis of *MdGA2ox7* and *MdGA20ox2* in different apple varieties.** (a, b) Expression levels of *MdGA2ox7* (a) and *MdGA20ox2* (b) in standard‑type and spur‑type varieties. (c, d) Expression levels of *MdGA2ox7* (c) and *MdGA20ox2* (d) in dwarfing and standard rootstocks. Data are means ± SD of three independent experiments. The significance test was carried out by one-way analysis of variance (ANOVA) followed by Tukey’s test, and different lowercase letters above the bars indicate significant differences among branches of different apple varieties (*p* < 0.05).

**FIGURE S6.** **Effects of** ***MdGA2ox7* on apple plant height and GA content.** (a) Phenotypic photograph of *MdGA2ox7* knockdown transgenic apple plants. (b) Relative expression level of *MdGA2ox7*. (c) Plant height. (d) Internode length. Scale bar = 5 cm. (e) GA_3_ content. (f) GA_4_ content. Statistical significance was determined using Student’s *t*-test for comparisons between TRV2-EV and TRV2-*MdGA2ox7* plants, and asterisks (*) above the bar charts indicate significant differences (**p* < 0.05; ***p* < 0.01; ****p* < 0.001).

**FIGURE S7. Effects of *MdGA20ox2* on plant height and GA content.** (a) Subcellular localization of MdGA20ox2. Scale bar = 20 μm. (b‑d) *MdGA20ox2* overexpression in transgenic *Arabidopsis*. (b) Plant photographs. (c) Plant height. (d) Internode length. In (c) and (d), one-way analysis of variance (ANOVA) followed by Tukey’s test was used for multiple comparisons, and different lowercase letters above the bar charts indicate significant differences among Col-0 and various *MdGA20ox2*-OE transgenic plants (*p* < 0.05). (e‑j) Effects of *MdGA20ox2* knockdown in apple on plant height and GA content. (e) Plant photographs. (f) Relative expression level of *MdGA20ox2*. (g) Plant height. (h) Internode length. (i) GA_3_ content. (j) GA_4_ content. In figures (f), (g), (h), (i), and (j), statistical significance was determined using Student’s *t*-test for comparisons between TRV2-EV and TRV2-*MdGA20ox2* plants, and asterisks (*) above the bar charts indicate significant differences (**p* < 0.05; ***p* < 0.01; ****p* < 0.001). (k‑m) Effect of exogenous GA_3_ treatment on *MdGA20ox2* knockdown apple. (k) plant photographs. (l) plant height. (m) internode length. For (b), (e), and (k), scale bars = 5 cm. In figures (l) and (m), asterisks (*) above the bars indicate significant differences between different treatments within the same line (**p* < 0.05; ***p* < 0.01; ****p* < 0.001); different uppercase and lowercase letters above the bars represented significant differences among different lines under the same treatment condition at the *p* < 0.05 level.

**FIGURE S8. Sequence alignment of MdSLR1b with rice SLR1.**

**FIGURE S9. Alignment analysis of *MdKNOX15* promoter sequences in different apple germplasms.** (a) Sequence alignment of the promoter region upstream of the *MdKNOX15* start codon obtained by PCR cloning. (b) Sequence alignment of the promoter region upstream of the *MdKNOX15* start codon from some published apple genomes.

**Table S1. Primers used for vector construction.**

| **Primer name** | **Primer sequence (5′→3′)** |
| --- | --- |
| MdKNOX15-V2F | GTGAGTAAGGTTACCGAATTCATGGAGGAAATGTACGGATTGC |
| MdKNOX15-V2R | GAGACGCGTGAGCTCGGTACCGTCGGATTTCGTCCAAGAAAC |
| MdGA2ox7-V2F | GTGAGTAAGGTTACCGAATTCATGGTGTTTGTGACCAAATCAG |
| MdGA2ox7-V2R | GAGACGCGTGAGCTCGGTACCACTCTTCTGCGTCATTTCCAAAA |
| MdGA20ox2-V2F | GTGAGTAAGGTTACCGAATTCATGGCTGTTGAGTGCATGATC |
| MdGA20ox2-V2R | GAGACGCGTGAGCTCGGTACCCATGCAGCGGTGAGCG |
| MdSLR1b-V2F | GTGAGTAAGGTTACCGAATTCATGAAGAGAGAACACTGCCATAG |
| MdSLR1b-V2R | GAGACGCGTGAGCTCGGTACCTTATCGTCGAGGACTCCCCA |
| MdKNOX15-2300F | ACGAGCTCGGTACCATGGAGGAAATGTACGGATTGCA |
| MdKNOX15-2300R | CATGGTGTCGACTCTAGATTCATTTGTAAAGAATGGTCCAGTAAG |
| MdGA20ox2-2300F | ACGAGCTCGGTACCATGGCTGTTGAGTGCATGATC |
| MdGA20ox2-2300R | CATGGTGTCGACTCTAGACAGTTTTTGGTTGGTTTTCTGTTGAAC |
| MdSLR1b-2300F | ACGAGCTCGGTACCATGAAGAGAGAACACTGCCATAG |
| MdSLR1b-2300R | CATGGTGTCGACTCTAGACTCCGTTGAGCTCGCCA |
| MdSLR1b-HA-R | CATGGTGTCGACTCTAGATCAAGCGTAATCTGGAACATCGTATGGGTACTCCGTTGAGCTCGCCA |
| FLAG-F | CTAGAGATTACAAGGACGATGACGACAAATGACTGCA |
| FLAG-R | GTCATTTGTCGTCATCGTCCTTGTAATCT |
| MdSLR1b-1301-F | GACTCTTGACCATGAAGAGAGAACACTGCCATAG |
| MdSLR1b-1301-R | CCTTTACTAGTCAGATCCTCCGTTGAGCTCGCCA |
| MdGA2ox7-0390F | CTGCAGGTCGACGGATCCCCGGGGGCTACATAAGAGCAACTCCACTG |
| MdGA2ox7-0390R | GGTGGACTCCTCTTAGAATTCTGTCACTTGTTATCAATATGTTTTATCTAATGTGTCT |
| MdGA20ox2-0390F | CTGCAGGTCGACGGATCCCCGGGTCAATAGAATATGTCTCTTCTCTTGTGATGC |
| MdGA20ox2-0390R | GGTGGACTCCTCTTAGAATTCTTTTGCATGAACAGAAAATGTATGTGAGT |
| MdKNOX15-His-F | CTTTAAGAAGGAGATATACCATGGAGGAAATGTACGGATTGCA |
| MdKNOX15-His-R | TCAGTGGTGGTGGTGGTGGTGCTCGAGTTCATTTGTAAAGAATGGTCCAGTAAG |
| MdSLR1b-His-F | CTTTAAGAAGGAGATATACCATGAAGAGAGATCACCGCGATAGC |
| MdSLR1b-His-R | TCAGTGGTGGTGGTGGTGGTGCTCGAGCTCGGTTGCGCTCGCCA |
| MdKNOX15-GST-F | CTGGTTCCGCGTGGATCCGAGGAAATGTACGGATTGCAATCA |
| MdKNOX15-GST-R | CGATGCGGCCGCTCGAGTCATTCATTTGTAAAGAATGGTCCAGTAAGATTATCC |
| MdSLR1b-AD-F | GCCATGGAGGCCAGTGAATTCAAGAGAGAACACTGCCATAGCTC |
| MdSLR1b-AD-R | CAGCTCGAGCTCGATGGATCCTCACTCCGTTGAGCTCGCC |
| MdKNOX15-BD-F | ATGGCCATGGAGGCCGAATTCTCATCGGATGAGGATTTCAG |
| MdKNOX15-BD-R | CCGCTGCAGGTCGACGGATCCTCATTCATTTGTAAAGAATGGTCCAGTAAG |
| MdKNOX15-cLUC-F | GATCGCCGTGTCTAGAATGGAGGAAATGTACGGATTGCA |
| MdKNOX15-cLUC-R | GCTTGATATCGAATTCTTCATTTGTAAAGAATGGTCCAGTAAG |
| MdSLR1b-nLUC-F | GGCGGCCGCTCTAGAATGAAGAGAGAACACTGCCATAG |
| MdSLR1b-nLUC-R | CAGCCCGGGGGATCCCTCCGTTGAGCTCGCCA |
| MdKNOX15-YFPN-F | CCTACTAGTGGATCCATGGAGGAAATGTACGGATTGCA |
| MdKNOX15-YFPN-R | CGGGAGCGGTACCTTCATTTGTAAAGAATGGTCCAGTAAG |
| MdSLR1b-YFPC-F | CGCCACTAGTGGATCCATGAAGAGAGAACACTGCCATAG |
| MdSLR1b-YFPC-R | CGGGAGCGGTACCCTCCGTTGAGCTCGCCA |
| proMdKNOX15-F | CACTGCCGTCGATGTTTTTATCATG |
| proMdKNOX15-R | CAACGTATACAACCCCACAAGC |

**Table S2. Primers used for RT-qPCR and ChIP-qPCR.**

| **Gene Name** | **Primer name** | **Primer sequence (5′→3′)** |
| --- | --- | --- |
| *MdKNOX15* | MdKNOX15-qF | TTGCAATCAACGGCCGACTA |
|  | MdKNOX15-qR | AGTGCAGAAGGCTGTGGTAA |
| *MdSLR1b* | MdSLR1b-qF | GTGGGATGGAAGTTGGCTCA |
|  | MdSLR1b-qR | GACAACACTTTGTCCACCGC |
| *MdGA2ox7* | MdGA2ox7-qF | ATTCCCCTCCCTTTCTACCAAGTAC |
|  | MdGA2ox7-qR | GTTATTTCATTCCGTCGAGGTTC |
| *MdGA20ox2* | MdGA20ox2-qF | TGGTGACACCTTCATGGCTC |
|  | MdGA20ox2-qR | AGCAGCATCGACCATGTGAA |
| *MdActin* | MdActin-qF | CCATTGGAGCAGAGCGTTTCC |
|  | MdActin-qR | GGAACATGGTCGAACCACCACTA |
| *MdMDH* | MdMDH-ChIP-qF | GACCATACATTCAGGGCACTTG |
|  | MdMDH-ChIP-qR | TGGCAAGTGACTGGGAATGAG |
| *MdGA2ox7* | MdGA2ox7-ChIP-qF | ACTTTTTCTGGTGATGCCCAC |
|  | MdGA2ox7-ChIP-qR | ACATAACACAATGACGTGCCG |
| *MdGA20ox2* | MdGA20ox2-ChIP-qF | CACGTTCGTTATCAAATATTCGTTGC |
|  | MdGA20ox2-ChIP-qR | GTTGCCCTAACAGTCACCATCTG |
